# Growth of a newly isolated oleaginous microalgal strain (*Asterarcys* sp. RA100) in oil produced water and its potential for biodiesel production

**DOI:** 10.1371/journal.pone.0325759

**Published:** 2025-06-17

**Authors:** Raeid M. M. Abed, Huda Al Battashi, Thirumahal Muthukrishnan, Hamzah Al-Bartamani, Mahmood Al-Hinai, Maryam Al-Baluchi

**Affiliations:** 1 Biology Department, College of Science, Sultan Qaboos University, Al Khoud, Sultanate of Oman; Khalifa University, UNITED ARAB EMIRATES

## Abstract

The prospects of using produced water (PW), a by-product of oil extraction, as a cultivation medium to not only grow microalgae but also generate value-added by-products has not been much investigated. This study demonstrates the ability of a newly isolated microalga, *Asterarcys* sp. RA100, for growth in PW and biodiesel production. Although the used PW was slightly alkaline (pH < 10), nutrient deficient and high in boron content, *Asterarcys* sp. RA100 exhibited good growth with phosphate, and nitrate, reaching optimal growth at 1% salinity, 25°C, 150 rpm, and 4000–8000 Lux LED light intensity. To test for its scalability in a greenhouse, *Asterarcys* sp. RA100 exhibited areal productivity of 10.3 ± 0.5 g m^–2^ day^–1^. Lipid accumulation in *Asterarcys* sp. RA100 reached 27.0 ± 5.1% of dry weight when grown in PW. The resulting fatty acids methyl esters (FAME) displayed properties aligning with international biodiesel standards. The FAME profiles showed elevated contents of palmitic acid (C16:0), elaidic acid (C18:1n9t), stearic acid (C18:0) and palmitoleic acid (C16:1n7C). This study demonstrates the immense potential of *Asterarcys* sp. RA100 to grow in PW and to serve as valuable feedstock for biodiesel production thereby, providing an eco-friendly method to re-use PW and sustain future energy demands.

## Introduction

Produced water (PW), which is a major by-product of oil extraction and drilling processes, has gained increasing attention in recent years for its potential application as a growth medium to cultivate microalgae [1–4]. PW is generated in large volumes reaching up to 250 million barrels per day worldwide [5], with approximately 6–8 barrels of PW produced for every barrel of extracted oil. PW is often nutrient-deficient and includes varying concentrations of hydrocarbons, heavy metals, toxic chemicals and suspended solids [3,5]. Despite the inherent contamination levels, several microalgal strains belonging to *Dunaliella*, *Chlorella*, *Scenedesmus*, *Nannochloropsis*, *Parachlorella*, and Chlorophyceae have demonstrated an ability to grow in PW [1,2,4–6]. This is mainly attributed to the notion that microalgae can easily adapt to different environments [7]. The production of PW in large amounts and the availability of non-arable lands adjacent to sites of PW outflow elevate the overall value of PW-based microalgal cultivation from an eco-friendly and economical perspective, without depleting existing freshwater resources. However, the selection of a suitable strain of microalgae for large scale growth and production in PW depends on several factors, including PW characteristics (e.g., salinity, hydrocarbon level, and heavy metal concentration), site specificity (e.g., temperature and light intensity), feasibility of upscaling, and economic value.

After successfully growing microalgae in PW at a large scale, the question of what can be done using PW-cultivated algal biomass warrants further investigation on the valuable by-products that can be obtained thereafter. Although microalgae offer a rich source of proteins and nutrients, there are serious health concerns associated with using the algal biomass growing in contaminated PW for animal and human feed. Therefore, bioprospecting PW-cultivated microalgae for biofuels remains as a better alternative. Several species of microalgae belonging to *Dunaliella*, *Chlorella*, *Botryococcus*, *Chlamydomonas*, *Thalassiosira*, and *Phaeodactylum*, could accumulate lipids up to 30–40% of their cell dry weight, and some strains increased their lipid content under stressful conditions [7,8]. So far, only oleaginous strains belonging to *Dunaliella*, *Chaetoceros* and *Chlorella* with total lipid content ranging from 13 to 40% weight per dry weight were able to grow in PW [4,5,9,10]. Growth of other oleaginous microalgae in PW has rarely been reported. Indeed, PW from different geographical locations can vary in their attributes including level of oil contamination, heavy metal content, and salinity, thus making it difficult for a single algal strain to grow in different types of PW.

The main objective of this study was to test the ability of a newly isolated microalgal strain to grow in PW, and at the same time to check its potential to accumulate lipids and produce biodiesel. The growth of the isolated strain was tested in the PW, and at different nutrient concentrations, salinities, temperatures, light intensities, and culture agitation speeds to find out the optimum growth conditions. The feasibility of scale-up and large-scale cultivation in outdoor conditions was evaluated. Lipid accumulation of the isolated strain was assessed under laboratory conditions and thereafter under nutrient and salinity stress. Subsequently produced biodiesel, after esterification of extracted lipids, was examined for its qualities in comparison to petroleum-derived diesel.

## Materials and methods

### Isolation and identification of the microalgal strain

A new microalgal strain was isolated from Wadi Al Khoud, Muscat, Oman (23° 35’ 19.32’‘ N, 58° 7’ 34.32’‘ E), a desert stream rich in dense freshwater algal mats. Pieces of the algal mats were inoculated into liquid BG-11 medium composed of (per liter): NaNO_3_ (1.5 g), MgSO_4_.7H_2_O (75 mg), CaCl_2_ (36 mg), Citric acid (6 mg), Ferric ammonium citrate (11 mg), and trace elements (1 mL). The trace metal solution contained (per liter): H_3_BO_3_ (2.86 g), MnCl_2_.4H_2_O (1.81 g), ZnSO_4_.7H_2_O (0.22 g), Na_2_MoO_4_.2H_2_O (0.39 g), CuSO_4_.5H_2_O (80 mg), and Co(NO_3_)_2_.6H_2_O (50 mg). The medium was autoclaved at 121°C for 15 min. After autoclaving, 1 mL of pre-sterile stocks of K_2_HPO_4_ (30.5 g L^–1^), Na_2_CO_3_ (20 g L^–1^), 10 ml of NaHCO_3_ (84.01 g L^–1^) and TES Buffer (229 g L^–1^, adjusted to pH 8.2 with NaOH) were added. Cultivation was carried out using 100 mL BG-11 medium in 250 mL flask in the laboratory at 100 rpm on an orbital shaker (Stuart SSL1, Germany) at 25°C under light intensity of 5000 Lux (white LED lamps) with light/dark cycle of 12:12 h. Strain purification was done using microscope-based micromanipulation and serial dilution methods until a unialgal culture (not axenic) was obtained. The morphological features of the isolated algal strain were studied using light microscopy (Olympus CX-23), scanning electron microscope (SEM), and transmission electron microscope (TEM). The microalgal strain was identified by sequencing its 18S rRNA gene. DNA was extracted from algal biomass using Qiagen DNeasy Kit (Hilden, Germany) according to the manufacturer’s instructions, with slight modifications to the cell lysis steps. Algal DNA samples were sent to the sequencing facility at Macrogen Ltd. (Seoul, Korea) for sequencing. PCR amplification was performed using the universal primers NS1 (5’-GTAGTCATATGCTTGTCTC-3’) and NS8 (5’-TCCGCAGGTTCACCTACGGA-3’) at an annealing temperature of 50°C [11]. Amplified genes were sequenced using BigDye® Terminator v3.1 Cycle Sequencing Kit (Applied Biosystems, USA) and an ABI PRISM 3730XL Analyzer (ThermoFisher Scientific, USA). The obtained sequence was Blasted using the database and engine available in the NCBI GenBank. The creation of a phylogenetic tree was carried out using MEGA 11 software.

### Growth optimization

PW was freshly collected from a constructed wetland in Southern Oman, more specifically from the evaporation ponds, which contain treated PW after passing through 350 ha of the wetland (for exact location see [12]). The wetland is currently treating over 115,000 m^3^ of PW from oilfields every day [2,13–15]. PW constituents were analyzed using an inductive coupled plasma optical emission spectrometer (ICP-OES) (8000 DV, Perkin Elmer, US) and ion chromatography (IC) (Metrohm, US). Due to its nutrient deficiency, PW was supplemented with 1X BG-11 concentration of nitrate and phosphate in the form of NaNO_3_ (1.5 g L^–1^), and K_2_HPO_4_ (0.03 g L^–1^), respectively. The growth of the microalgal strain in PW was tested under different nutrient and incubation conditions (for details see S1 Table). The experiments were conducted over 15 days using 160 ml glass serum bottles, each containing 40 ml medium inoculated with 3% of the algal culture. The algal inoculum was prepared before the optimization experiments by growing the culture in PW amended with 1X nitrate and phosphate, and then splitting the batch culture into three replicates, ensuring same initial cell densities as measured by a spectrophotometer at 680 nm wave length. The bottles were incubated at 25°C and 150 rpm shaker (IKA incubator shaker KS 4000 *i*) under light intensity of 5000 Lux (white LED) with light/dark cycle of 12:12 h or conditions were changed depending on the examined parameter. Samples (1 ml each) were withdrawn every 3 days and growth was spectrophotometrically measured (Thermo Scientific, Germany) at 680 nm wavelength. All experiments were conducted in three biological replicates (i.e., three separate cultures). The study examined the effects of the following parameters, with the specific incubation conditions for each experiment detailed in S1 Table:

1) Presence of 1X nitrate and 1X phosphate separately and when mixed
2) Different concentrations of nitrate (0, 0.5, 1, 1.5 and 2X) in the presence of 1X phosphate
3) Different concentrations of a mixture of nitrate and phosphate (0, 1, 2, 4, 6, and 8X each)
4) Presence of vitamins (0.5 ml)
5) Presence of trace elements (1 ml)
6) Presence of iron (1 ml)
7) Salinity (0, 1, 2, 3, 4 and 5%), adjusted by diluting PW using distilled water or allowing it to naturally evaporate under indirect sunlight until the required salinity was obtained.
8) Temperature (15, 25 and 45°C)
9) Light intensity (laboratory LED light: 1078, 4000, 6000, and 8000 Lux)
10) Shaking regime (static, 100, 150 and 200 rpm).

The vitamin solution was composed of 200 mg L^–1^ of thiamine HCl, 1 ml of Biotin (Vitamin H) stock (1.0 g L^–1^ dH_2_O), and 1 ml of cyanocobalamin (vitamin B_12_) stock (1.0 g L^–1^ dH_2_O). Trace metals and iron solution concentrations were used similar to those used in BG-11 medium.

### Scalability and outdoor cultivation

Prior to outdoor cultivation, the microalgal strain was up-scaled from a volume of 0.25 L to 1 L using 10% inoculum (v/v) and PW amended with the major nutrients (1X nitrate and phosphate) under laboratory conditions (see above). Duplicate flasks were maintained, and one was used to initiate the starter culture (4 L) for outdoor cultivations. Preliminary trials of cultivation exposed to direct sunlight showed lack of growth of the microalgal strain, hence subsequent cultivation attempts were performed under indirect sunlight. Using filtered, nutrient-amended PW, approximately 4 L of algal culture was established in an open plastic aquarium (0.05 m^2^), under indirect sunlight (max. intensity of 2300 Lux) outside the laboratory (max. 25–27°C). Starting inoculum of 10% (v/v) was used to initiate cultivation ensuring same optical density (OD) values measured spectrophotometrically at 680 nm as that measured during the previous scale-up to 1 L. To substitute the shaking conditions previously maintained in the laboratory, a submersible mixing pump was placed at one end of the aquarium (water velocity of 0.5–1 m s^–1^) to facilitate a homogenous suspension of the algal strain. The salinity of the PW was maintained at 1% by regularly replenishing the evaporated water with appropriate amounts of distilled water. Growth was spectrophotometrically estimated by withdrawing triplicate samples (2 ml per replicate) of the microalgal culture, and measuring the OD at 680 nm. To verify for air-borne contamination, culture samples were microscopically examined at 1000X magnification (Nikon, Japan). The time point at which to feed nutrients was determined based on the changes in OD values, i.e., constant OD values indicating the time to add nutrients (1X nitrate and phosphate). After 21 days, the microalgal culture scale-up for 8 L was done by first harvesting 0.8 L of the previously grown culture, and then adding 7.2 L of nutrient-amended fresh PW (1X nitrate and phosphate) to the same aquarium. The culture growth and quality were monitored over time using spectrophotometry and light microscopy, respectively along with salinity maintenance as previously mentioned.

Outdoor cultivation of the algal strain in a total volume of 35 L of PW was established under greenhouse conditions (maximum air temperature of 28–29°C, and maximum sunlight intensity of 20000 Lux). An open plastic aquarium (0.172 m^2^) containing submersible mixing pumps (water velocity at ~1.5 m s^–1^) and aeration tubes (continuous air flow rate of 3.8 m s^–1^) was used to maintain a culture depth of 0.22 m. Transparent cling films were used to cover the aquarium to avoid air-borne pest or dust contamination. The algal growth during cultivation was frequently monitored during the first week and at regular intervals of time thereafter using triplicate samples withdrawn from the aquarium (5 ml per replicate). Microscopic examination of culture was used to monitor for the presence or absence of contamination over time. Following spectrophotometric measurements, the algal biomass from each replicate was harvested (5000 rpm, 15 min), rinsed with distilled water and dried in a hot-air oven at 50°C for 1–2 h or until constant weight was reached. Dried biomass weight values were used to calculate the volumetric and areal productivity of the microalgal strain as follows: The dry biomass concentration (g L^–1^) in each replicate culture sample was estimated by dividing the dry weight (g) per replicate by the volume of each replicate (L). Volumetric productivity (V) expressed as g L^–1^ day^–1^ was calculated by dividing the difference between the final and initial dry biomass concentrations (g L^–1^) by the time required to reach the final concentration (days). Areal productivity (A) expressed as g m^–2^ day^–1^ was calculated by multiplying the volumetric productivity (V) with the total volume of the culture (35 L) and dividing by the area occupied by the aquarium (0.172 m^2^).

### Lipid extraction and quantification

Lipid extraction from the isolated microalgal strain was performed using chloroform/methanol as described in [16]. The extraction was conducted using 50 mg dry algal biomass. Lipid accumulation was evaluated during growth of the isolated microalgal strain in PW and BG-11 (2% salinity). For the growth of the strain, a low-cost photobioreactor was fabricated using 5 L transparent plastic bottles. The system includes an aeration setup with air pumps to ensure efficient gas exchange, and each bottle was fitted with a port for nutrient and inoculum additions. The strain was cultivated in two of these fabricated photoreactors under ca. 5000 Lux (white LED light) for 12:12 h light: dark cycle with continuous aeration at room temperature (ca. 25°C). Samples (2 ml) were withdrawn every two days for microalgal growth estimation using a spectrophotometer. At different stages of the algal growth, 200 ml of culture were harvested to determine the lipid content.

The effect of nitrate and phosphate deprivation on lipid accumulation by the microalgal strain was tested. The culture growing in 5 L PW amended with 1X nitrate and phosphate for 10 days was harvested by centrifugation at 10,000 rpm at 25°C for 5 min., and the biomass was re-suspended in different flasks. Four nutrient stress conditions were tested in the presence and absence of either phosphate or nitrate or both (i.e., + P–N, + N–P, + P + N, and ‒P‒N). The effect of salinity changes on lipid accumulation was also tested. The culture was re-suspended in PW medium at the salinities 0, 1, 3, and 5%. The flasks were incubated in a shaker incubator (IKA KS 4000*i*) at 25°C and 120 rpm, under LED light illumination with 12:12 h light/dark cycles. Lipid content was determined after 3 and 5 days of incubation using the optimum protocol.

### Transesterification and biodiesel characterization

Following lipid extraction from the samples incubated at 2% salinity at 25°C with 1X nitrate and phosphate addition, lipid samples were subjected to transesterification. The samples were mixed with 1 ml of NaOH in methanol (0.5 M) and heated at 100°C for 15 min in a water bath. After cooling in a cool water bath, 2 ml of BF_3_ methanol-complex was added and mixed into the solution, which was subsequently heated once more in a water bath at 100°C for 5 min. The solution was then cooled once again in water followed by the addition of 1 ml of hexane and 2 ml of ddH_2_O. The solution was then mixed, spun down and the hexane phase was collected and diluted with hexane as needed for fatty acid methyl ester (FAME) analysis using gas chromatography (GC-MS).

The FAME composition of the produced biodiesel was determined through GC-MS analysis (GCMS-QP2010 Ultra Shimadzu) equipped with mass spectrometry detection and using a 100 m × 0.25 mm × 0.2 µm film thickness fused silica capillary column. For each analysis 1 µm of produced biodiesel in hexane was injected at a 10.0 split ratio with helium being used as a carrier gas at a flow. The injector temperature was kept at 250°C, the detector temperature at 270°C, and the oven temperature was set to 50°C with a rate of increase of 4°C/ min with a 15-min hold at 250°C. Peaks of fatty acids were determined and quantified using Agilent’s analysis software and by comparing the sample spectra to an established library. Octanoic acid was used as an internal standard for quantification. Theoretical values for the biodiesel characteristics were calculated using the FAME composition through previously established mathematical models [17]. These models allowed us to determine the predicted values for cetane number (CN), saponification value (SV), iodine value (IV), degree of unsaturation (DU), cold filter plugging point (CFPP), cloud point (CP), and higher heating value (HHV).

### Statistical analysis

Statistical analysis of the data was conducted using independent-sample T test to compare the means of two variables (control versus vitamin, trace elements, or iron additions). One way analysis of variance (ANOVA) was used to analyze the means of three or more groups depending on the experimental factors. This was followed by Tukey’s HSD Post Hoc multiple comparison analysis to determine the significance (*p* < 0.05) between different treatments at different time points using IBM SPSS statistical package (version 21).

## Results

### Nutrient analysis of PW

The PW from the constructed wetlands had a salinity of 1 ± 0.2%, and a slightly alkaline pH of 10 ± 0.3 (Table 1). The PW was deficient in nitrite, phosphate and ammonium, but contained ca. 8.2 ± 2 mg L^–1^ nitrate. Sulphate was detected at the concentration of 1241 ± 91 mg L^–1^. The concentrations of sodium and chloride were higher than other salts like fluoride, potassium, magnesium and calcium. PW contained several metals, with boron showing the highest concentration of 23.9 ± 0.6 mg L^–1^.

**Table 1:** Physico-chemical characteristics of the PW from the constructed wetland.

Parameters	PW
Salinity (%)	1 ± 0.2
pH	10 ± 0.3
Dissolved O_2_ (mg/L)	3.36 ± 0.3
*Nutrients*	*(mg/L)*
Nitrate	8.2 ± 0.2
Nitrite	ND
Phoshate	ND
Sulphate	1241 ± 91
Bromide	38.5 ± 1.9
Chloride	8243 ± 420
Flouride	10.5 ± 0.6
Ammonium	ND
Potassium	82.5 ± 4.6
Sodium	6953 ± 334
Magnesium	268 ± 56
Calcium	364 ± 238
*Elements/ Heavy Metals*	*(mg/L)*
Aluminium	1.1 ± 0.3
Copper	1.9 ± 0.4
Manganese	0.3 ± 0.03
Silicon	11.8 ± 0.1
Boron	23.9 ± 0.6
Iron/Barium/Phosphorus/Zinc	ND
Arsenic/Cadmium/Nickel	ND

ND: not detected.

### Identification of the microalgal strain

**Fig 1:**
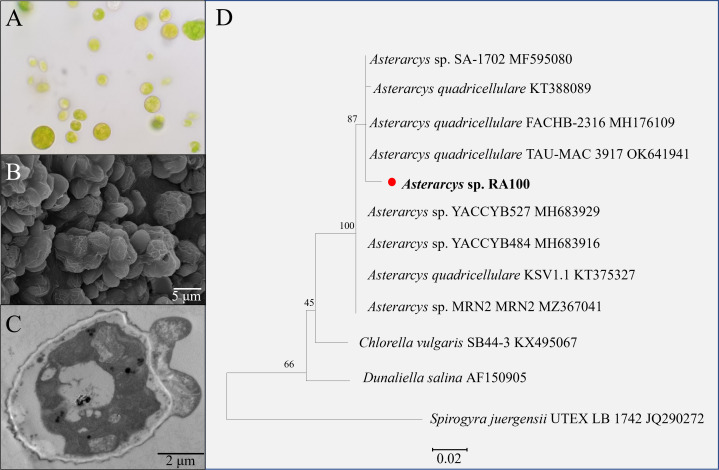
Morphological and phylogenetic analyses of the oleaginous alga *Asterarcys* sp. RA100, isolated from Wadi Al Khoud, Oman. Light microscopy (A), SEM image (B), TEM image (C) and a phylogenetic tree based on neighbour-joining (D) of *Asterarcys* sp. RA100.

Light microscopy showed that the isolated microalgal strain was unicellular, non-motile, and spherical in shape (Fig 1A). Cell diameter ranged from approximately 9 µm to 15 µm while the cells appeared yellow or orange to green depending on the growth stage (Fig 1A). The cell shape ranged from being spherical to ellipsoidal when observed using SEM and TEM. The latter showed presence of intracellular oil globules as observed under light microscope (Fig 1B, C). Primarily, a planktonic mode of growth was exhibited with occasional flocculation or biofilm formation depending on potential stress or physiological changes in the culture. Based on 18S rRNA sequencing, the strain was identified as *Asterarcys* sp. RA100 owing to its phylogenetic similarity (98% sequence similarity, sequence length of 607 bp) to *Asterarcys quadricellulare (*Accession number: KT388089), and to other unclassified species of *Asterarcys* (YACCYB527, YACCYB484, and SA-1702, Fig 1D).

### Growth optimization of *Asterarcys* sp. RA100

**Fig 2:**
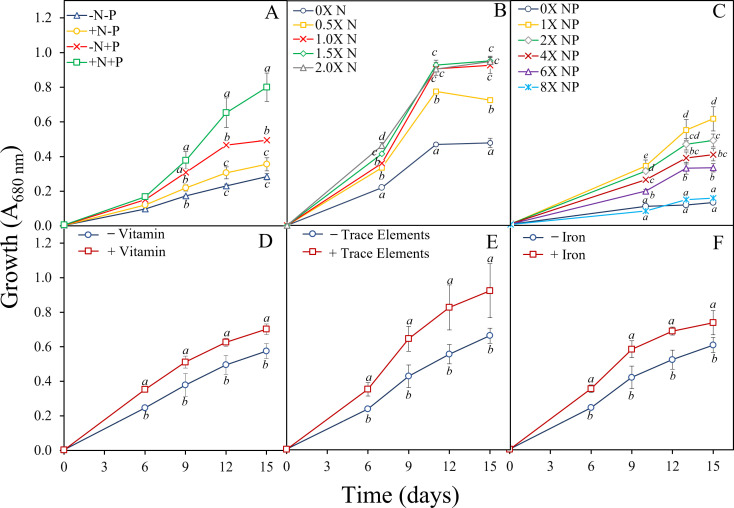
Growth of *Asterarcys* sp. RA100 at different nutrient loadings (A‒F) using PW as a growth medium. The experiment was designed to test the effect on growth of A) addition of 1X nitrate (N: corresponding to 1.5 g L^–1^) and 1X phosphate (P: corresponding to 0.03 g L^–1^) separately and when combined; B) addition of different concentrations of nitrate, in presence of 1X phosphate; C) addition of different concentrations of nitrate and phosphate: and D-F) with and without vitamins, trace elements and iron. Unamended PW was used as a control (-N-P). Growth was monitored spectrophotometrically measured at 680 nm. All experiments were conducted in three biological replicates. Common alphabetic superscripts indicate no significant difference using Tukey’s test within each time point.

*Asterarcys* sp. RA100 exhibited the highest level of growth with the addition of both nitrate and phosphate (Fig 2A; *p* ≤ 0.017). The addition of either nitrate or phosphate alone also increased growth, but with significantly higher growth levels in the case of phosphate (*p* ≤ 0.05). The incubation of *Asterarcys* sp. RA100 at different nitrate concentrations showed similar growth pattern in all concentrations, except at 0X and 0.5X nitrate (Fig 2B), while an exposure to increasing concentrations of both nitrate and phosphate exhibited a decrease in growth (Fig 2C). The highest growth was detected in the presence of 1X nitrate and phosphate concentration. The addition of vitamins, trace elements and irons significantly increased the growth of *Asterarcys* sp. RA100 compared to PW amended with only nitrate and phosphate (Fig 2D, E and F; *p* ≤ 0.03, 0.04 and 0.02 respectively).

**Fig 3:**
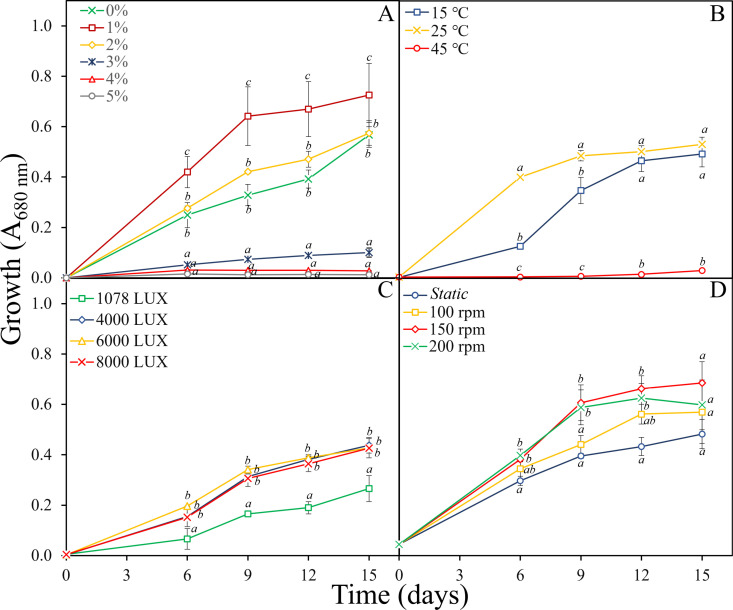
Growth of *Asterarcys* sp. RA100 at different salinities (A), temperatures (B), light intensities (C) and shaking regimes (D) using PW as a growth medium. Growth was monitored spectrophotometrically measured at 680 nm. All experiments were conducted in three biological replicates. Common alphabetic superscripts indicate no significant difference using Tukey’s test within each time point.

The growth of *Asterarcys* sp. RA100 decreased with increasing salinity, with maximum growth detected at 1% salinity after 15 days (Fig 3A). The growth at 0% and 2% salinities was comparable, but there was no growth detected at 4 and 5% salinity. The incubation of *Asterarcys* sp. RA100 at different temperatures showed an optimum growth of the strain at 25°C (Fig 3B), and no growth at 45°C. The algal growth at 15°C was significantly lower (*p* ≤ 0.005) than at 25°C in the first 9 days of incubation but reached comparable levels thereafter (*p* > 0.26). When *Asterarcys* sp. RA100 was exposed to different light intensities, the growths was comparable in indoor conditions at light intensities between 4000 and 8000 Lux, but significantly lower (*p* < 0.0001) at 1078 Lux (Fig 3C). Shaking of the culture had also an influence on growth. The growth of *Asterarcys* sp. RA100 increased with increasing shaking, with maximum growth reached at 150–200 rpm.

### Scale-up of *Asterarcys* sp. RA100 in outdoor conditions

**Fig 4:**
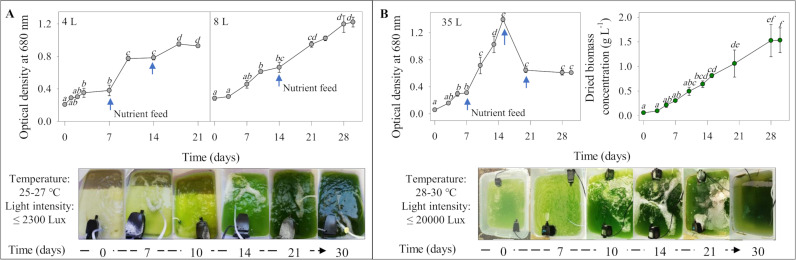
Scale-up of *Asterarcys* sp. RA100 in an 8 L aquarium under greenhouse settings (A), then in an 35L aquarium under outdoor conditions (B). Environmental conditions in both cases are mentioned next to photographs showing the development of the culture over a period of 30 days. The culture growth and quality were monitored over time using spectrophotometry and light microscopy. Arrows indicate the frequency of nutrient addition to maintain growth. Volumetric and areal productivities were calculated from the produced biomass. Common alphabetic superscripts indicate no significant difference using Tukey’s test within each time point.

The outdoor cultivation of *Asterarcys* sp. RA100 using nutrient-amended PW under indirect sunlight established initially at the 4 L scale did not show very significant changes in growth during the first week based on the measured OD values (Fig 4A, p > 0.05). However, the first nutrient feed of 1X nitrate and phosphate after one week stimulated algal growth and thereafter, a weekly supply of nutrient feed was required until 21 days of cultivation (Fig 4A). When scaled up to 8 L in the same aquarium, the nutrient feed frequency decreased to once in two weeks over a cultivation period of 30 days (Fig 4A). At the 35 L scale under greenhouse conditions, a weekly feed of nutrients was required to maintain culture growth over a period of 15 days (Fig 4B). The average volumetric and areal productivity of *Asterarcys* sp. RA100 was 0.05 ± 0.003 g L^–1^ day^–1^ and 10.3 ± 0.5 g m^–2^ day^–1^, respectively. After 15 days, there were no significant increases in algal growth based on the OD values but an insignificant increase in the total dried biomass concentration. The onset of grazer contamination followed by algal biomass flocculation leading to a non-homogenous culture suspension was microscopically detected thereafter (Fig 4B).

### Lipid accumulation

**Fig 5:**
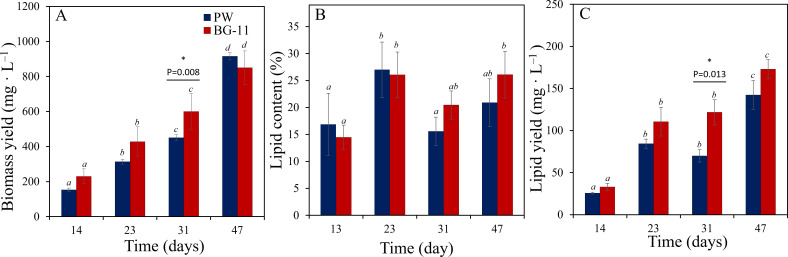
Algal biomass (A), lipid content in % (B) and lipid yield in mg L^‒1^ (C) of *Asterarcys* sp. RA100, which grew on 1X nitrate and phosphate amended PW in 5 L fabricated photobioreactors in the laboratory under LED lights for 12:12 of dark: light cycle. Common alphabetic superscripts indicate no significant difference using Tukey’s test within each time point. Stars indicate the time points at which different incubations exhibit statistically significant difference (p < 0.05).

The growth of *Asterarcys* sp. RA100 in the fabricated 5 L photobioreactor showed a uniform and steady buildup of biomass with time in both BG-11 and PW media (Fig 5A). *Asterarcys* sp. RA100 accumulated higher biomass, but not significant in BG-11 than in PW medium, but the biomass yield in both media was comparable at the end of the incubation (Fig 5A). Estimation of lipid accumulation in *Asterarcys* sp. RA100 showed the ability of the strain to accumulate similar amounts of lipids in both media. While the maximum lipid content of ca. 26% was detected after 23 days of incubation, the highest lipid yield, when factoring in the amount of harvested biomass was estimated (Fig 5B and 5C).

**Fig 6:**
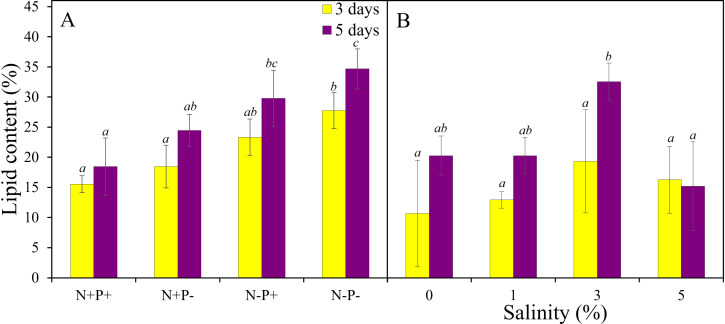
The effect of nutrient deprivation (A) and salinity stress (B) on lipid accumulation in *Asterarcys* sp. RA100 growing in PW. Incubations were done with (+) and without (-), nitrate (N) and phosphate (P), and the salinities 0, 1, 3, and 5%. All experiments were conducted in three biological replicates. Common alphabetic superscripts indicate no significant difference using Tukey’s test within each time point.

When *Asterarcys* sp. RA100 was subjected to stressful conditions of nutrient deprivation and higher salinities, lipid accumulation increased to above 30% (Fig 6). In the nutrient deprivation experiment, the culture supplemented with both nitrate and phosphate (+N + P) displayed the lowest lipid content of 16 and 18% whereas the culture that lacked both nutrients displayed the highest lipid content of 28 and 35% after 3 and 5 days of incubation, respectively (Fig 6A; *p* ≤ 0.014). There was no significant difference between the lipid content at 0% and 1% salinity, but exposure to 3% salinity increased lipid content to reach 33% after 5 days of incubation (Fig 6B).

### FAME analysis and biodiesel characterization

The FAME profiles of the lipids extracted using chloroform/methanol from BG-11 and PW grown microalgal biomass were comparable (Table 2). The most dominant FAMEs in the extracted lipids were C18:1n9t (36% and 33.6% in PW and BG-11 respectively), C16:0 (19.2% and 21%), C18:0 (10.2% and 11.2%), and C18:2 (10.2% and 12.4%). The majority of fatty acids in the FAME profiles were monounsaturated fatty acids (42.6% and 41.1% in PW and BG-11 respectively) followed by unsaturated fatty acids (30.9% and 33.7%) and lastly polyunsaturated fatty acids (23.5% and 23.3%).

**Table 2:** Comparison of FAME composition of *Asterarcys* sp. RA100 cultivated in PW and BG-11 with other *Asterarcys* spp. grown in artificial medium and/or wastewater.

Strain	*Asterarcys* sp. RA100		*A. quadricellulare* (MZ323982) [61]	*A. quadricellulare* KNUA020 [35,60]		*A. quadricellulare* [67]	
Medium	PW	BG-11	BG-11	BG-11	wastewater	wastewaters	
C11:0	ND	ND	0.3	ND	ND	ND	ND
C12:0	ND	ND	0.1	12	ND	0.9	ND
C14:0	ND	ND	3.4	ND	3.5	6.2	5.6
C15:0	ND	ND	ND	ND	0.6	0.9	ND
C16:0	19.2	21.0	27.4	15.3	24.7	35.6	35.7
C17:0	ND	ND	0.52	ND	ND	ND	ND
C18:0	10.2	11.2	1.0	ND	1.6	27.2	28.9
C20:0	ND	ND	0.4	ND	ND	ND	ND
C21:0	ND	0.5	0.5	ND	ND	ND	ND
C22:0	0.3	ND	0.0	ND	ND	ND	ND
C24:0	ND	ND	0.0	ND	ND	ND	ND
C28:0	1.2	1.0	ND	ND	ND	ND	ND
⅀**SFA**	**30.9**	**33.7**	**33.5**	**27.3**	**30.4**	**70.8**	**70.2**
C15:1	ND	ND	0.3	ND	ND	ND	ND
16:1n7c	6.6	7.5	0.5	6.8	2.4	ND	ND
C17:1	ND	ND	3.14	ND	ND	ND	ND
C18:1n9t	36.0	33.6	18.1	ND	23.4	26.1	23.8
C20:1	ND	ND	0.8	ND	ND	ND	1.4
⅀**MUSFA**	**42.6**	**41.1**	**22.8**	**6.8**	**25.8**	**26.1**	**25.2**
C16:2n4	8.0	5.8	ND	ND	0.6	ND	ND
C16:3	ND	ND	ND	ND	1.1	ND	ND
C16:4	ND	ND	ND	14.8	7.9	ND	ND
C18:2	10.2	12.4	22.8	ND	3.7	3.3	3.6
C18:2ω6	5.3	5.1	ND	3.6	ND	ND	ND
C18:3 alpha	ND	ND	17.3	41.2	27.2	ND	ND
C18:4	ND	ND	ND	ND	3.2	ND	ND
C20:3c	ND	ND	0.42	ND	ND	ND	ND
C20:3t	ND	ND	0.17	ND	ND	ND	ND
C20:4	ND	ND	0.3	ND	ND	ND	1.2
C22:6	ND	ND	0.4	ND	ND	ND	ND
⅀**PUSFA**	**23.5**	**23.3**	**41.4**	**59.6**	**43.8**	**3.3**	**4.8**
⅀**UFA**	**66**	**64**	**64**	**66**	**70**	**29**	**30**
**SFA/UFA**	**0.47**	**0.52**	**0.52**	**0.41**	**0.44**	**2.41**	**2.34**

*ND: Not detected; SFA: saturated fatty acids; MUFA: monounsaturated fatty acids; PUFA: polyunsaturated fatty acids

The microalgae grown in PW and BG-11 yielded biodiesel with cetane values of 61 and 59, respectively, which are greater than that required by the EN 14214 standard (Table 3). The sapomification value (SV) of produced biodiesel from both media were comparable at 179 and 186 in PW and BG-11 respectively. The cloud point (CP) and the cold filter plugging points (CFPP) of biodiesel produced from PW cultures were slightly lower than for the biodiesel produced from BG-11. The predicted iodine values (IV) and densities of the produced biodiesel were lower than the standard values. The degree of unsaturation of the produced biodiesels reached 74–76 whereas the predicted higher heat values were 35–36 (Table 3).

**Table 3:** Comparison of biodiesel lipid and biodiesel properties of *Asterarcys* sp. RA100 growing in PW and BG-11 with other algal cultures.

Strains	Medium	Volume (L)	Salinity (%)	Biomass (mg/L)	Lipid (%)	Biodiesel Properties								Reference
						CN	SV	CP	CFPP	IV	Density	DU	HHV	
Standard Value (EN 14214)						>51	NA	NA	NA	≤120	0.86-0.9	NA	NA	
*Asterarcys* sp. RA100	PW	5	2	915	27	61	179	5.1	5.6	72	0.8	74	35	This study
*Asterarcys* sp. RA100	BG11	5	2	850	26	59	186	6	7.7	74	0.8	76	36	This study
*Asterarcys quadricellulare*	75% Municipal Wastewater	0.15	NA	1440	25	58.7	184	5.8	−9.1	77	0.8	84	36	[64]
*Asterarcys quadricellulare* KNUA020	Municipal Wastewater	0.5	0.5	~640	17	44.7	179	NA	−7	131	0.9	102	NA	[57]
*Asterarcys quadricellulare*	Pre-Chlorinated Wastewater	0.8	0.42	460	21	65.5	207	14.1	43.2	32	0.9	32	39	[65]
*Scenedesmus obliquus*	Nitrate deprived BG-11	0.65	0	550	48	59	195	NA	−2.5	66	NA	77	NA	[61]
*Chlorella pyrenoidosa*	Nitrate deprived BG-11	0.65	0	420	55	44	198	NA	−0.8	133	NA	120	NA	[61]
*Chlamydomonas reinhardtii*	Fish farm wastewater	10	NA	~130	NA	60	194	NA	−4.5	94	NA	NA	40	[63]
*Coelastrella* sp. M-60	BG-11	0.1	3	NA	37	56	195	NA	44.3	85	NA	92	NA	[62]
*Micractinium* sp. M-13	Nitrogen deprived BG-11	0.1	0	NA	27	63	191	NA	4	53	NA	58	NA	[62]

**DU:** Degree of Unsaturation, **Density**: (g/cm^3^) **SV:** Saponification Value (mg/g), **IV:** Iodine Value (gI_2_/100 g fat), **CN:** Cetane number, **CFPP:** Cold Filter Plugging Point (°C), **CP:** Cloud Point (°C), **HHV:** Higher Heating Value (MJ/kg), **NA:** Not Availabe.

## Discussion

Our data suggests that PW constitutes a suitable growth medium for our oleaginous strain *Asterarcys* sp. RA100 after amendment with nitrate and phosphate under indoor and outdoor conditions. The major advantages of using PW in Oman as a growth medium for algae is its availability in large amounts and proximity to large non-arable lands next to oil fields. The PW obtained not only from Oman but also from other locations in Australia and United States seems to lack the essential nutrients nitrate and phosphate for algal growth [3,18,19]. Nevertheless, PW contains several ions and heavy metals that could support algal growth. For instance, calcium provides a structural support to algal cell walls and serves as a secondary messenger during cell stress [3]. Previous reports have shown that majority of algae require aluminum, copper, and manganese as micronutrients, while boron was shown to improve the qualities of the algal cell wall in case of C*hlorella vulgaris* and *Microcystis aeruginosa* [3,20–22]. Although boron concentration was the highest among all detected heavy metals (i.e., ca. 24 mg L^–1^), this concentration did not seem to harm the growth of *Asterarcys* sp. R100. In fact, the ability of *Asterarcys* sp. RA100 to exhibit similar growth in PW to that in BG-11 medium without any addition, except for nitrate and phosphate, indicates the potential of PW to be used as a valuable nutrient medium containing essential metals and metalloids.

### Growth characteristics of *Asterarcys* sp. RA100

Our growth experiments clearly demonstrated that addition of nitrate and phosphate to PW is a prerequisite before use as a cultivation medium for algae. Phosphate seems to have a more significant effect on the growth of *Asterarcys* sp. RA100 than nitrate, which could be attributed to the inherent P-deprivation in PW (both PO_4_ and P are ND as per Table 1) in comparison to N, which is already present as nitrate in minor quantities (i.e., 8.2 ± 2 mg L^–1^). It is well established that phosphate plays a key role in multiple biological functions in algae, including, but not limited to, ATP metabolism, nucleic acid metabolism, and cell signaling [23–25]. It should be kept in mind though that higher concentrations of phosphate can inhibit the growth of algae due to the formation and precipitation of Ca_3_PO_4_, with the available calcium in PW, particularly at higher pH (> 8), which could consequently lead to algal flocculation [26]. On the other hand, nitrate is known to be essential to various functional components of algae such as proteins, nucleic acid, enzymes, chlorophyll, and energy production [27–28]. Despite that, higher concentrations of nitrate (from 1.5 to 3 g L^-1^) had little effect on the biomass production of *Asterarcys* sp. R100. This is congruent with some previous reports, which showed that some algae like *Asterarcys* sp. BTA9034, and *Chlorella kessleri* did not show much changes in growth with nitrate concentrations ranging from 0.375 to 6 g L^-1^ [25,29,30]. The addition of vitamins, trace elements, and iron has also promoted the growth of *Asterarcys* sp. RA100, however, were excluded from follow-up experiments because of their high cost [31].

Growth of *Asterarcys* sp. RA100 at different salinities and temperatures suggests that this strain is brackish and mesophile. The ability of the strain to grow at salinities below 2% is consistent with the original environment from where the strain was isolated [32]. Higher salinities are known to affect various biochemical and physiological processes in algae, including growth and lipid production [4]. On the other hand, the failure of *Asterarcys* sp. RA100 to grow at 45°C could be due to the drastic effect of high temperatures on protein structure and enzyme activity [8,33,34]. However, the growth of *Asterarcys* sp. RA100 optimally at 25°C is consistent with the growth of other *Asterarcys* spp. such as *A. quadricellulare*, which grew best at temperatures between 25 and 37°C [8,35,36]. Although our strain was not tested within the range between 25–45°C; it was indeed able to grow at temperatures higher than 25°C, as could be observed during the scale-up experiments. The growth of *Asterarcys* sp. RA100 exposed to light intensities up to 8000 Lux revealed its adaptability to varying light availability. Despite the fact that exposure to high light intensities results in PSII photo-damage leading to photo-inhibition, reduction of biomass productivity, and eventually culture crash [37–39]. Optimal growth of other *Asterarcys* spp. have been previously reported at light intensities up to 10000 Lux [8,39,40].

### Scale-up of algae in outdoor conditions

The successful scale-up of *Asterarcys* sp. RA100 from 0.25 L in the laboratory (25°C, LED light intensity of 5000 Lux, constant shaking at 100 rpm) to 35 L under greenhouse conditions (max. 25–27°C, natural sunlight intensity of max. 20000 Lux, pump-mediated culture mixing) reflects the scalability potential of our strain. Reports about the scale-up of microalga in PW are quite limited [2,41]. Strains belonging to *Cyanobacterium* sp. and *Scenedesmus* sp. have demonstrated their ability to grow in PW at a large scale under open-air conditions exposed to direct sunlight [2,41]. However, this was not the case for our strain despite its origin from an environment exhibiting high temperature and light intensity. Although it is not possible to extrapolate our laboratory-based growth optimization results to real-world scenarios, exposure to light intensity beyond a limit has been shown to cause serious impacts on the structural integrity, growth, and development of microalgae [2,42]. Hence, if we were to cultivate *Asterarcys* sp. RA100 at a large scale under outdoor conditions in the future, the construction of sheltered areas will be required to maintain ambient temperature and light intensity during the summer, unlike the winter season when conditions would be comparatively more favorable for microalgal growth. Furthermore, the frequency of nutrient feed will also need to be taken into account while establishing an outdoor cultivation system. Previous studies that focused on the outdoor cultivation of microalgae in PW have indicated a nutrient feed frequency of once in every three or four days to maintain growth [2,41], unlike every week in case of our strain. Changes in nutrient feed frequency can be partially attributed to the type of microalgae grown, the total volume of PW used and the field conditions. It must be kept in mind that microalgal strains requiring frequent nutrient feeds can incur a substantial addition to the overall cultivation expenses on an annual basis.

The areal productivity of *Asterarcys* sp. RA100 obtained in our study was lower to that reported in previous attempts using brackish PW as a cultivation medium [2,41]. A maximum areal productivity of 16 g m^–2^ day^–1^ was demonstrated during the outdoor cultivation of *Cyanobacterium* sp. in a total volume of 700 L of fertilizer-amended PW with a salinity of 1.2% after 23 days [41]. On the other hand, investigations that used different cultivation media (F/2 and modified BG-11 with the salinity of 3.5 and 1%, respectively) and incubation periods (15–33 days) to grow *Tetraselmis* sp., *Chlorella* sp. and *Monoraphidium* sp. reported comparable productivity values (10–11 g m^–2^ day^–1^) to our study [42,44]. The inherent challenges faced during outdoor cultivation such as grazing, and potential PW volume reduction owing to temperature fluctuations thereby leading to sudden salinity shifts may contribute to overall changes in the culture productivity from time to time [2,5,41–48]. Hence, to determine the actual productivity range of a selected strain of microalga, further investigations would be required keeping in mind not only the possible changes that can occur regarding the strain physiology but also the surrounding environmental conditions during outdoor cultivation on an annual basis.

### Lipid content in *Asterarcys* sp. RA100

The lipid analysis of *Asterarcys* sp. RA100 qualifies it to be a potential candidate for biodiesel production using PW as a growth medium. This is evident from the ability of *Asterarcys* sp. RA100 to accumulate 27.0 ± 5.1% and 26.1 ± 4.2% lipids in PW and BG-11, respectively. Previously investigated *Asterarcys* species exhibited variable levels of lipid accumulation, depending on the cultivation medium and the incubation conditions (see Table 4). While some oleaginous *Asterarcys* species accumulated up to 44% lipids of their dry weight, others such as *Asterarcys* sp. SCS-1881 had comparable amounts to our strain [8,25,49–52]. The lipid content of other algal strains grown in PW varied between 18–48% per dry weight. For instance, the lipid content of our strain was similar to that of *Chlorella* sp. USU080 (26%), but lower than that of *Dunaliella tertiolecta* (40%) and the polyculture of *Parachlorella kessleri* and *Cyanobacterium aponinum* (48%). Despite the lower lipid content in our strain, the lipid yield (in g L^–1^), when considering the biomass per volume of culture, was still higher (0.040 g L^–1^) than the polyculture of *Parachlorella kessleri* and *Cyanobacterium aponinum* (0.029 g L^–1^) [4,5,25,49–52]. Furthermore, recent studies on mutagenesis in microalgae have shown success in increasing lipid yields [53].

**Table 4:** Lipid content of *Asterarcys* sp. RA100 growing in PW in comparison with other *Asterarcys* spp. grown in artificial medium (BG-11 and BB medium) and other microalgal cultures growing in PW.

Strain	Waste source	Culture volume	Cultivation conditions	Salinity	Biomass	Biomass productivity	Lipid content	Lipid yield	Ref.
		(L)		%	(g/L)	(g/L/d)	%	(mg/L)	
*Asterarcys* sp. RA100	PW	5	Temp.: 25°C	2	0.915	0.04	27	247	This study
			Light intensity: 5000 Lux (12:12 light-dark cycle)						
*Asterarcys* sp. (BTA9034)	BG-11	0.25	Temp.: 28°C	0	_	_	6–11%	_	[25]
			Light intensity: 44 μmol/m^2^/s (14:10 light dark cycle).						
*Asterarcys quadricellulare* KNUA020	BG-11		Temp.: 25°C pH 7.0.	0	_	_	16	_	[35]
			Air flow rate: 2 L/min						
			Light intensity: 70 μmol/m^2^/s (16:8 light-dark cycle)						
*Asterarcys* sp. SCS-1881	BG-11	0.6	Temp.: 25°C	0	3.7	_	26	966	[50]
			Light intensity:100 μmol/m^2^/s (24:0 light-dark cycle)						
*Asteracys* sp.	BG-11	0.1	Temp.: 25°C	0		_			[49]
			Light intensity: 900 μmol/m^2^/s (12:12 light-dark cycle)						
			Auotroph (A)		A 0. 883		A 33	295	
			Mixotrophic mode (500 mg/L glucose) (M)		M 4.348		M 40	1739	
*Asterarcys quadricellulare*	BB medium	0. 1	Temp.: 37°C	0	1.29	_	44	571	[9]
			Light intensity: 250 μmol/m^2^/s (4:10 light-dark cycle)						
*Asterarcys quadricellulare*	BB medium	2	Temp.: 24ºC	0	0.463-0.567	_	20	_	[48]
			Light intensity: 133 μmol/m^2^/s (12:12 light-dark cycle)						
			Growth mode: mixotrophic conditions (0.1 g/L glucose)						
*Dunaliella tertiolecta*	Coal seam gas PW	4	Light intensity: 2000 Lux	1	0.4	0.0497	22	88	[11]
Polyculture (*Cyanobacterium aponinum* and *Parachlorella kessleri*)	PW	0.25	Temp. 24°C	6	0.35	0.0291	31	108	[6]
			Light intensity: 100 μmol/m2/s	6	_	_	48	_	[6]
*Dunaliella tertiolecta*	PW	0.25	Temp.: 24°C	6	_	_	40	60	[5]
			Light intensity: 100 μmol/m^2^/s						
*Chaetoceros gracilis*	PW	0.45	Temp.: 24°C	2.58	1.065	0.1184	18	191	[10]
			Light intensity: 300 μmol/m^2^/s						
*Chlorella* sp. USU080	PW	0.45	Light intensity: 300 μmol/m^2^/s	2.58	1.286	0.1285	26	338	[10]

The increase in lipid accumulation in *Asterarcys* sp. RA100 when incubated under nutrient deprivation is consistent with previous reports [54,55]. Microalgae tend to modify their lipid biosynthesis pathways and increase their lipid content when nutrients become limited in an attempt to reserve carbon sources [54–56]. Similarly, salinity stress induced lipid accumulation in *Asterarcys* sp. RA100 as a result of accompanied salinity-dependent physiological and biochemical changes [4,57–59]. Salinity stress has been previously shown to increase lipid production in algae such as *Monoraphidium* sp. QLY-1, in which lipid content increased from 35% at 0% salinity to 47% at 2% salinity [59].

### FAME and biodiesel characteristics

Lipid extraction from PW and BG-11 grown microalgal biomass produced FAME profiles containing the most common fatty acid in biodiesel such as C16:0 (palmitic acid), C18:0 (stearic acid), C18:1n96 (oleic acid), and C18:2 (linolenic acid) in similar percentages to previously derived biodiesel from different feedstocks [50,60,61]. When compared to other species of *Asterarcys*, our strain had lower ratios of C16:0, but greater ratios of C18:2, C16:1n7c, and C18:1n9t [50,60]. The high ratios of monounsaturated fatty acids (e.g., C16:1n7c and C18:1n9t) in biodiesel have been known to provide oxidative stability and better cold flow behavior [61,62]. The degree of unsaturation (DU) of lipids in our species was noticeably lower than that of previously studied microalgae, which can be attributed to the lower proportion of polyunsaturated fatty acids present [60]. Despite this, the average ratio of saturated to unsaturated fatty acids remained similar due to the greater percentage of mentioned monounsaturated fatty acids [60–61]. Higher ratios of saturated to unsaturated fatty acids are typically sought after for biodiesels, due to high saturation, resulting in higher CN and HHV, which are both integral aspects of biodiesels.

The CN, SV, IV were found to be within recommended values by international standards (EN 14214 and ASTM D6751). The CN values of the produced biodiesel, i.e., 61 and 59 in PW and BG-11 respectively, were higher than both the CN value minimum of 51 required according to the EN 14214 standard and those found in biodiesels previously produced from microalgae, which is likely a result of greater proportion of long chain fatty acids [60,63–66]. Biodiesels with higher CN have lower ignition delay, improving the quality of the biodiesel. The SV of the produced biodiesel was found to be at 179 and 186 mg KOH/g in PW and BG-11, respectively, which falls below the recommended limit of 370 mg KOH/g by the ASTM D6751 standard. The low predicted SV in the produced biodiesel indicates a lower amount of triglycerides than has previously been reported for similarly produced biodiesels [63–67]. The CP and CFPP were predicted to be above the −20–0 °C requirement for CFPP set by EN 14214, thus indicating that the produced biodiesel would have poor performance in non-tropical conditions. This is expected as cold flow properties are determined by degree of unsaturation, chain size among other factors [62,68]. The poor cold flow properties are likely a result of the low proportion of polyunsaturated fatty acids present, which have been known to benefit cold flow properties [69]. The predicted IV of our produced biodiesels was both under the standard maximum value according to EN 14214, which is indicative of the magnitude of unsaturation. IV is typically used as an indicator for susceptibility to oxidation and as a result a lower value has been associated with chemical stability. The predicted density of our biodiesel was lower than the requirement by the EN 14214 standard, which might be correctable with better optimizations of biodiesel production from *Asterarcys* sp. RA100. Additionally, the HHV is lower than those previously seen in biodiesels produced from microalgae, indicating a lower amount of energy released per volume of biodiesel when compared to similarly produced biodiesels, which is likely a result of the lower proportion of saturated fatty acids present in our biodiesel [65,69,70]. Despite this, *Asterarcys* sp. RA100 shows promise as a feedstock for biodiesel according to international standards with better optimizations to the biodiesel production process.

## Conclusion

In conclusion, our study demonstrated the potential use of nitrate and phosphate-amended oil PW as a growth medium to propagate microalgae at a large scale. Our isolate *Asterarcys* sp. RA100, serves as a good example, whose growth in PW generated large biomass that was successfully used as a valuable feedstock for biodiesel production. The use of such technology could provide an eco-friendly method to re-use PW and sustain energy demands in the future. From an economical perspective, the use of PW for algal growth, instead of freshwater, and the availability of non-arable land adjacent to sites of PW outflow, will contribute to the decrease of the overall cost of PW-microalgal cultivation for biodiesel production. Nevertheless, future research is still required to make this approach more cost-effective, to optimize the growth of *Asterarcys* sp. RA100 in runways under real world conditions, and to optimize methods for algae harvesting, lipid extractions and biodiesel production.

## Supporting information

S1 Table Details of growth optimization experiments of *Asterarcys* sp. RA100 at different nutrient concentrations and incubation conditions.
(XLSX)
